# *Echinacea purpurea* root extract mitigates hepatotoxicity, genotoxicity, and ultrastructural changes induced by hexavalent chromium via oxidative stress suppression

**DOI:** 10.1007/s11356-024-32763-7

**Published:** 2024-03-08

**Authors:** Fatma M. El-Demerdash, Mustafa M. Karhib, Nora F. Ghanem, Mohamed M. Abdel-Daim, Raghda A. El-Sayed

**Affiliations:** 1 Department of Environmental Studies, Institute of Graduate Studies and Research, Alexandria University, 163 Horreya Avenue, P.O. Box 832, Alexandria, Egypt; 2 Department of Medical Laboratory Techniques, College of Health and Medical Technologies, Al-Mustaqbal University College, 51001 Hillah, Babylon, Iraq; 3 Department of Zoology, Faculty of Science, Kafr ElSheikh University, Kafr ElSheikh, Egypt; 4 Department of Pharmaceutical Sciences, Batterjee Medical College, Pharmacy Program, P.O. Box 6231, Jeddah, 21442 Saudi Arabia; 5 Pharmacology Department, Faculty of Veterinary Medicine, Suez Canal University, Ismailia, 41522 Egypt

**Keywords:** *Echinacea purpurea* root extract, Chromium (VI), Oxidative stress/inflammation/apoptosis, Histopathology/ultrastructural examination, Hepatotoxicity

## Abstract

Environmental and occupational exposure to hexavalent chromium (CrVI) is mostly renowned as a possible hepatotoxic in mammals. *Echinacea purpurea* (L.) Moench, a phenolic-rich plant, is recurrently used for its therapeutic properties. Therefore, this investigation was done to explore whether *E. purpurea* (EP) root extract would have any potential health benefits against an acute dose of CrVI-induced oxidative damage and hepatotoxicity. Results revealed that GC–MS analysis of EP root extract has 26 identified components with a significant amount of total phenolic and flavonoid contents. Twenty-four Male Wistar rats were divided into four groups: control, EP (50 mg/kg BW/day for 21 days), CrVI (15 mg/kg BW as a single intraperitoneal dosage), and EP + CrVI, respectively. Rats treated with CrVI displayed a remarkable rise in oxidative stress markers (TBARS, H_2_O_2_, PCC), bilirubin, and lactate dehydrogenase activity, and a marked decrease in enzymatic and non-enzymatic antioxidants, transaminases, and alkaline phosphatase activities, and serum protein level. Also, CrVI administration induced apoptosis and inflammation in addition to histological and ultrastructural abnormalities in the liver tissue. The examined parameters were improved significantly in rats pretreated with EP and then intoxicated with CrVI. Conclusively, EP had a potent antioxidant activity and could be used in the modulation of CrVI-induced hepatotoxicity.

## Introduction

A naturally occurring element that can be found in the Earth’s crust is chromium (Callender 2003). Both natural and anthropogenic sources contribute to its release into the environment, with industrial discharges accounting for the majority of the total (Duruibe et al. 2007). The broad distribution of CrVI in the environment, as well as the release of wastewater discharge containing chromium, may result in the contamination of soil and water (Sharma et al. 2022; Kader and Kalapuram 2017). CrVI is a significant contaminant that poses considerable risks to human health and the environment due to widespread industrialization. CrVI is mostly utilized in the chemical industry, dye manufacturing, wood preservation, leather tanning, electroplating, alloy manufacturing, and a variety of other uses (Mishra and Bharagava 2016). CrVI also affects the food chain, which puts human health in danger as it is a well-known inducer of several types of toxicity in different organs (Pereira et al. 2021; El-Demerdash et al. 2019; Mishra and Bharagava 2016; Marouani et al. 2017; Kim et al. 2018). In addition, animals exposed to CrVI suffer severe tissue damage, including lesions in various organs (Pereira et al. 2021; El-Demerdash et al. 2019, 2021a and 2021b). Its mode of action is thought to involve an increase in the formation of DNA adducts because of the surplus production of ROS and DNA harm (Khalil et al. 2013). CrVI can get into the bloodstream by a variety of mechanisms leading to liver dysfunction and tissue damage including cellular vacuolar degradation and necrosis (Mishra and Bharagava 2016; Soudani et al. 2013; Elshazly et al. 2016).

*Echinacea purpurea* (L.) Moench is an herbaceous perennial plant from the family Asteraceae with prolonged medicinal use in different countries (Barrett 2003). The immunostimulating activities of processed Echinacea output are related to its active phytochemical components like caffeic acid derivatives, alkamides, polysaccharides, melanins, and glycoproteins (Xu et al 2021).

Derivatives of caffeic acid particularly cichoric acid have a wide range of biological properties, inclusive of collagen protection, anti-hyaluronidase activity, antiviral activity, phagocyte activity promotion, and high antioxidant capacity (Barnes et al. 2005; Bauer and Wagner 1991; Pellati et al. 2004). Furthermore, *E. purpurea* possesses antifungal, antibacterial, anti-inflammatory, and anti-cancer activities (Paudel et al. 2023; Kabir et al. 2022; Hudson 2012; Gurley et al. 2012). Numerous investigations have shown that *E. purpurea* extracts efficiently protect against liver damage brought on by alcohol, chemical pollutants, and obesity (Xiao et al. 2013; Smalinskiene et al. 2007; Landmann et al. 2014). Therefore, this investigation was executed to assess the possible prophylactic role of *E. purpurea* root versus hepatotoxicity, oxidative toxicity as well as histopathological and ultrastructure variations induced by CrVI.

## Materials and methods

### Materials

Pure potassium dichromate (K_2_Cr_2_O_7_, 99%) was provided by Merck, Darmstadt, Germany. The rest of the chemicals were of analytical quality. *Echinacea purpurea* roots were obtained from the native shop in Alexandria, Egypt; recognized; and authenticated by the Botany Department, Faculty of Science, Alexandria University.

### Preparation of *E. purpurea* roots extract

Ethanol/water of *E. purpurea* roots extract was prepared according to Stanisavljeviü et al. (2009). *Echinacea purpurea* roots were properly cleaned before being weighed to about 1 g. An extract with a final ethanol level of 50% was created using 1 g of plant roots to 2 mL of solvent. The extraction solvent was made of 75% ethyl alcohol and 25% filtered water. The roots and solvent were properly blended before being left to macerate for 14 days, using a high-quality blender then preserved at 25 °C.

### GC/MS analysis

The chemical components of *E. purpurea* extract were recognized utilizing GC/MS version (5) 2009, a Thermo Scientific equipment containing a column (TG-5MS, 30mX0.32mmID) (Adams 2006). The ingredients of the extract were specified using mass fragmentation modality and the database of the mass spectrum of the National Institute of Standards and Technology (NIST, version 2).

### In vitro measurement of total phenolic content, total flavonoids, DPPH, ABTS^+^ antioxidant potency, and ferrous ion chelating capacity

The total phenolic contents (TPC), total flavonoids (TF), and antioxidant capacity using the DPPH and ABTS^+^ and ferrous ion chelating (FIC) capacity of *E. purpurea* root extract were measured according to the previously published methods by Singlenton and Rossi (1965), Chang et al. (2002), Brand-Williams et al. (1995), Rivero-Perez et al. (2007), and Gülçin (2005), respectively.

### Experimental layout

Male Wistar albino rats (150–170 g) were purchased from the animal house of the Faculty of Medicine, Alexandria University. The experimental protocol was authorized by the Local Ethics Committee and Animals Research of Alexandria University by following the National Institutes of Health guidelines for the care and use of Laboratory animals (NIH Publications No. 8023, revised 1978). The rats were retained in cages with a 12-h/12-h day/night cycle, 40–60% relative humidity, and indefinite access to water and feed. Following a week of acclimation, animals were classified randomly into four sets of six animals per each. The first group worked as a reference control (distilled water), and the second group gave oral supplementation of *E. purpurea* root extract (EP; 50 mg/kg BW daily for 3 weeks) (Mrozikiewicz et al. 2010). One intraperitoneal injection of hexavalent chromium (CrVI; 15 mg/kg BW) was administered to the third group (Sahu et al. 2014). The fourth group received EP orally for 3 weeks before the intraperitoneal injection of CrVI. Rats that had been treated with CrVI showed no signs of illness or mortality. After 48 h of CrVI treatment, animals were benumbed using isoflurane, cut down by cervical dislocation, then livers and blood were promptly collected.

### Preparation of serum and tissue samples

Cardiovascular puncture blood was drawn, let to clot for about 30 min at room temperature, and centrifuged at 3000 g for 15 min. Until it was required, sera were collected and preserved at − 20 °C. However, liver tissues were weighed, cut, and homogenized using sodium–potassium phosphate buffer (0.01 mol/L, pH 7.4), centrifuged (10,000 g; 4 °C; 20 min), and the supernatant fluids were retained for further testing.

### Assessment of biochemical parameters

The levels of thiobarbituric acid-reactive substances (TBARS), hydrogen peroxide (H_2_O_2_), and reduced glutathione (GSH) content were determined in liver homogenate. Also, the activities of superoxide dismutase (SOD; EC 1.15.1.1), catalase (CAT; EC 1.11.1.6), glutathione reductase (GR; EC 1.8.1.7), glutathione peroxidase (GPx, EC 1.11.1.9), and glutathione *S*-transferase (GST; EC 2.5.1.18), as well as aspartate aminotransferase (AST; EC 2.6.1.1), alanine aminotransferase (ALT; EC 2.6.1.2), and lactate dehydrogenase (LDH; EC 1.1.1.27), were quantified utilizing commercially available kits (Biodiagnostic, Egypt). However, alkaline phosphatase activity (ALP; EC 3.1.3.1) was measured according to Principato et al (1985). The technique of Reznick and Packer (1994) was applied to evaluate the protein carbonyl content (PCC). Serum bilirubin, albumin, and protein content were assessed (Walters and Gerade 1970; Lowry et al. 1951; Doumas et al. 1977), while the difference between total protein and albumin estimated the globulin concentration.

### Quantitative real-time PCR

The liver’s total RNA was taken away utilizing the Trizol reagent according to the technique of Yang et al. (2020a). Reverse transcription of RNA into cDNA was executed by applying a High Capability cDNA Reverse Transcription kit (Vazyme, Nanjing, China). The genes were coupled with the matching specific primers (Table 1). According to the instructions of the SYBR Green RT-qPCR Master Mix (Thermo Scientific, # K0221), the mRNA expression was quantitatively evaluated. Melting curve analysis was used to find PCR specificity. The relative mRNA level adjusted versus β-actin mRNA level was examined using a Bio-Rad CFX96 touch (Hercules, CA, USA). A comparative 2^−∆∆CT^ approach was used to do the calculations.

**Table 1:** Primers sequences for qPCR

Gene	Primer sequences
Housekeeping β-actin	F: AAGTCCCTCACCCTCCCAAAAG
	R: AAGCAATGCTGTCACCTTCCC
Inflammation	
IL-1β	F: CACCTCTCAAGCAGAGCACAG
	R: GGGTTCCATGGTGAAGTCAAC
TNF-α	F: GCATGATCCGCGACGTGGAA
	R: AGATCCATGCCGTTGGCCAG
NF-κβ	F: CCTAGCTTTCTCTGAACTGCAAA
	R: GGGTCAGAGGCCAATAGAGA
Apoptosis	
Bax	F: ACACCTGAGCTGACCTTG
	R:AGCCCATGATGGTTCTGATC
Bcl2	F: AGTACCTGAACCGGCATCTG
	R: CATGCTGGGGCCATATAGTT

### Histopathological investigation

Liver tissue was fixed in formalin-neutral buffer (10%), and dehydrated, then the obtained consecutive paraffin sections were stained with hematoxylin and eosin to assess histopathological variations (Bancroft and Steven 1990); then, each slide was photographed using a microscope (Olympus BX 41, Japan).

### Ultrastructural examination using transmission electron microscope

Fresh liver tissue sections (< 1 mm^3^) were fixed by immersing them immediately in “4% formaldehyde and 1% glutaraldehyde (4F:1G)” and phosphate buffer (pH 7.2) at 4 °C for 3 h, washing them using 0.1 M phosphate-buffered saline (PBS), fixing them using citric acid (1%) then rinsing them once more with 0.1 M PBS. The segments were dehydrated, soaked, embedded, polymerized, chopped, and sliced before being dyed by utilizing lead citrate and uranyl acetate. Liver ultrastructure sections were investigated using a transmission electron microscope (TEM) (Hitachi H-7650 Tokyo, Japan).

### Statistical analysis

The SPSS software was used to examine the means and standard errors of means (SEM) of the data from various groups (version 22, IBM Co., Armonk, NY). Following a one-way ANOVA, to compare the groups, a Tukey’s post hoc test was conducted. A *P*-value of 0.05 or less was significantly accepted.

## Results

### Phytochemistry and antioxidant properties of* E. purpurea* root extract

Twenty-six components were found with the aid of GC–MS including caffeic acid, cichoric acid, hexadecanoic acid, and linoleic acid (Table 2). *Echinacea purpurea* root extract has high levels of total flavonoids and phenolic contents, according to quantitative chemical analysis with values of 29.6 ± 0.12 mg GAE/mL and 0.23 ± 0.01 mg QE/mL, respectively. The results are consistent with the high antioxidant activity of the *E. purpurea* root extract, where an in vitro analysis showed to have a comparatively high capacity to scavenge DPPH with an IC_50_ value of 0.26 ± 0.01 g/mL, ABTS^+^ scavenging activity of 1.66 ± 0.06 g/mL, and FIC chelating capacity of 3.0 ± 0.02 mg/mL.

**Table 2:** Chemical composition of *E. purpurea* root extract obtained by GC–MS analysis

Number	Compound	RT (min)	Matching factor
1	2,3-Butanediol	14.97	1044
2	Hexadecanoic acid	16.56	1079
3	2-Methyl-4-pentenoic acid	16.83	1085
4	(E)-2-Hexenoic acid	18.83	1127
5	Verbenone	22.92	1215
6	Cichoric acid	28.54	1340
7	Copaene	30.49	1385
8	β-Caryophyllene	32.39	1430
9	Decanoic acid	33.91	1466
10	Epicubebol	35.47	1504
11	Malic acid	35.79	1512
12	Dihydroactinidiolide	36.83	1539
13	Trans-Nerolidol	38.09	1571
14	Spathulenol	38.79	1589
15	Caryophyllene oxide	39.03	1595
16	Dodecanoic acid	41.55	1662
17	Oplopanone	44.49	1755
18	Neophytadiene	46.35	1845
19	Myristic acid	46.53	1858
20	Pentadecanoic acid	47.78	1960
21	Linoleic acid	49.21	2109
22	Caffeic acid	49.62	2161
23	Phytol	49.85	2190
24	α-Tocopherol	57.56	3182
25	Stigmasterol	60.31	3353
26	β-Sitosterol	61.40	3416

### Lipid peroxidation, protein oxidation, and enzymatic and non-enzymatic antioxidants

The levels of protein carbonyl, TBARS, and H_2_O_2_ were significantly induced in rats injected with a single dose of CrVI accompanied by depletion in non-enzymatic antioxidant (GSH) and enzymatic antioxidants (SOD, CAT, GPx, GR, and GST) activities in liver homogenate. Animals supplemented with EP alone demonstrated a substantial drop in PCC, TBARS, and H_2_O_2_ levels and significant improvement in the GSH content and antioxidant enzyme activities as related to the control group. While animals administered with EP and then treated with CrVI displayed significant amendment in the assessed indices in comparison to the CrVI group (Table 3).

**Table 3:** Oxidative stress markers and antioxidant enzyme activity in liver of rats treated with *E. purpurea* (EP), chromium hexavalent (CrVI), and their combination

Parameters	Groups			
	Cont	EP	CrVI	EP + CrVI
TBARS (nmol/g tissue)	31.87 ± 1.26^c^	25.46 ± 0.93^d^	46.86 ± 1.39^a^	40.23 ± 1.59^b^
H_2_O_2_ (μmol/g tissue)	91.42 ± 3.21^c^	74.92 ± 2.91^d^	133 ± 5.09^a^	113 ± 3.98^b^
PCC (nmol/mg protein)	2.16 ± 0.063^c^	2.03 ± 0.064^c^	3.91 ± 0.104^a^	2.98 ± 0.115^b^
GSH (mmol/mg protein)	2.02 ± 0.062^b^	2.41 ± 0.088^a^	1.10 ± 0.047^d^	1.51 ± 0.060^c^
SOD (U/mg protein)	79.31 ± 2.58^b^	94.44 ± 3.30^a^	41.56 ± 1.74^d^	59.00 ± 2.09^c^
CAT (µmol/h/mg protein)	52.38 ± 1.95^b^	61.32 ± 1.96^a^	26.57 ± 0.97^d^	37.10 ± 0.93^c^
GPx (U/mg protein)	0.97 ± 0.036^b^	1.16 ± 0.034^a^	0.55 ± 0.020^d^	0.74 ± 0.029^c^
GR (U/mg protein)	1.21 ± 0.043^b^	1.40 ± 0.055^a^	0.75 ± 0.021^d^	1.00 ± 0.028^c^
GST (µmol/h/mg protein)	1.06 ± 0.039^b^	1.27 ± 0.042^a^	0.58 ± 0.018^d^	0.81 ± 0.024^c^

Values are expressed as means ± SE; *n* = 6/group. Means within a row not sharing common letters were significantly different, *p* < 0.05. Significant variations between groups are compared as follows: EP and CrVI are compared to control, while the EP + CrVI group is compared to the CrVI group

### Liver function biomarkers

In the current research, significant alterations in ALT, AST, LDH, and ALP activities and protein content, as well as serum biochemical parameters (total protein, albumin, globulin, and bilirubin) concentration were observed in the group intoxicated with CrVI compared to the control one. Some of the examined parameters were significantly influenced by EP administration alone. Moreover, rats given EP plus CrVI showed considerable improvement (Table 4).

**Table 4:** Effect of *E. purpurea* (EP), chromium hexavalent (CrVI), and their combination on the enzyme activities and protein content in rats

Parameters	Groups			
	Cont	EP	CrVI	EP + CrVI
Liver				
AST (U/mg protein)	126 ± 4.14^a^	121 ± 4.78^a^	76 ± 3.03^c^	96 ± 3.58^b^
ALT (U/mg protein)	152 ± 6.12^a^	148 ± 3.52^a^	98 ± 3.85^c^	121 ± 4.57^b^
LDH (U/mg protein)	1007 ± 36.29^c^	1054 ± 37.80^c^	1404 ± 47.52^a^	1197 ± 46.64^b^
ALP (U/mg protein)	385 ± 12.39^a^	414 ± 16.26^a^	243 ± 8.57^c^	306 ± 9.46^b^
Protein (mg/g tissue)	198 ± 5.95^a^	208 ± 7.46^a^	129 ± 5.50^c^	158 ± 5.17^b^
Serum				
Total protein (g/dL)	7.69 ± 0.248^a^	8.28 ± 0.325^a^	4.10 ± 00.145^c^	6.20 ± 0.193^b^
Albumin (g/dL)	5.17 ± 0.175^a^	5.42 ± 0.137^a^	2.91 ± 0.072^c^	3.99 ± 0.106^b^
Globulin (g/dL)	2.38 ± 0.094^a^	2.56 ± 0.049^a^	1.48 ± 0.037c	2.38 ± 0.080^ab^
Bilirubin (mg/dL)	0.742 ± 0.028^c^	0.690 ± 0.023^c^	1.046 ± 0.034^a^	0.886 ± 0.037^b^

Values are expressed as means ± SE; *n* = 6/group. Values within a row not sharing common letters were significantly different, *p* < 0.05. Significant variations are compared as follows: EP and CrVI are compared to control while the EP + CrVI group is compared to the CrVI group

### Inflammation

**Fig. 1:**
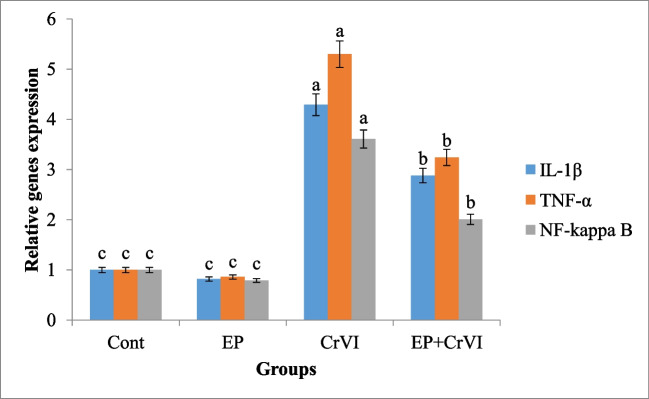
Effect of CrVI or/and EP treatment on the expression of liver inflammation-related genes; IL1β, TNFα, and NFκB as detected by qPCR. Data presented as fold change (means ± SEM, *n* = 6/group). Columns carrying different letters (a [the highest value]–c [the lowest value]) are significantly different at *P* < 0.05

The present qPCR results showed a significant rise in pro-inflammatory cytokines, NF-κB, TNFα, and IL1β mRNA levels, in the liver tissue of CrVI-intoxicated rats as related to the reference group. EP supplementation before CrVI treatment resulted in a considerable downregulation of all genes. The EP group’s expression was much lower than the other groups (Fig. 1).

### Apoptosis

**Fig. 2:**
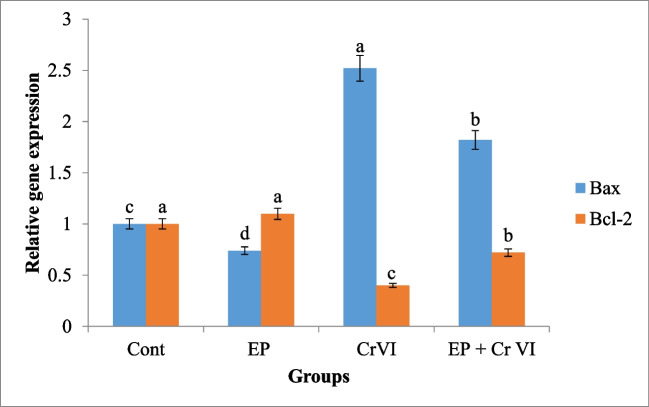
Effect of CrVI and/or EP treatment on the expression of liver apoptosis–related genes Bax and Bcl-2 as detected by qPCR. Data presented as fold change (means ± SEM, *n* = 6/group). Columns carrying different letters (a [the highest value]–d [the lowest value]) are significantly different at *P* < 0.05

CrVI-induced DNA damage in liver tissues could indicate apoptosis. To rule out this hypothesis, qPCR was performed to assess the expression of Bax, an apoptotic gene, and Bcl-2, an anti-apoptotic gene. A considerable overexpression in Bax and a decrease in Bcl-2 expression were observed in the liver tissue of rats given CrVI as related to the reference group. While rats that were administered with EP pretreatment and then received CrVI displayed a considerable decline in Bax and a rise in Bcl-2 as related to CrVI group. Administration of EP lonely resulted in a considerable drop in Bax expression and an insignificant change in Bcl-2 expression (Fig. 2).

### Histopathology

**Fig. 3:**
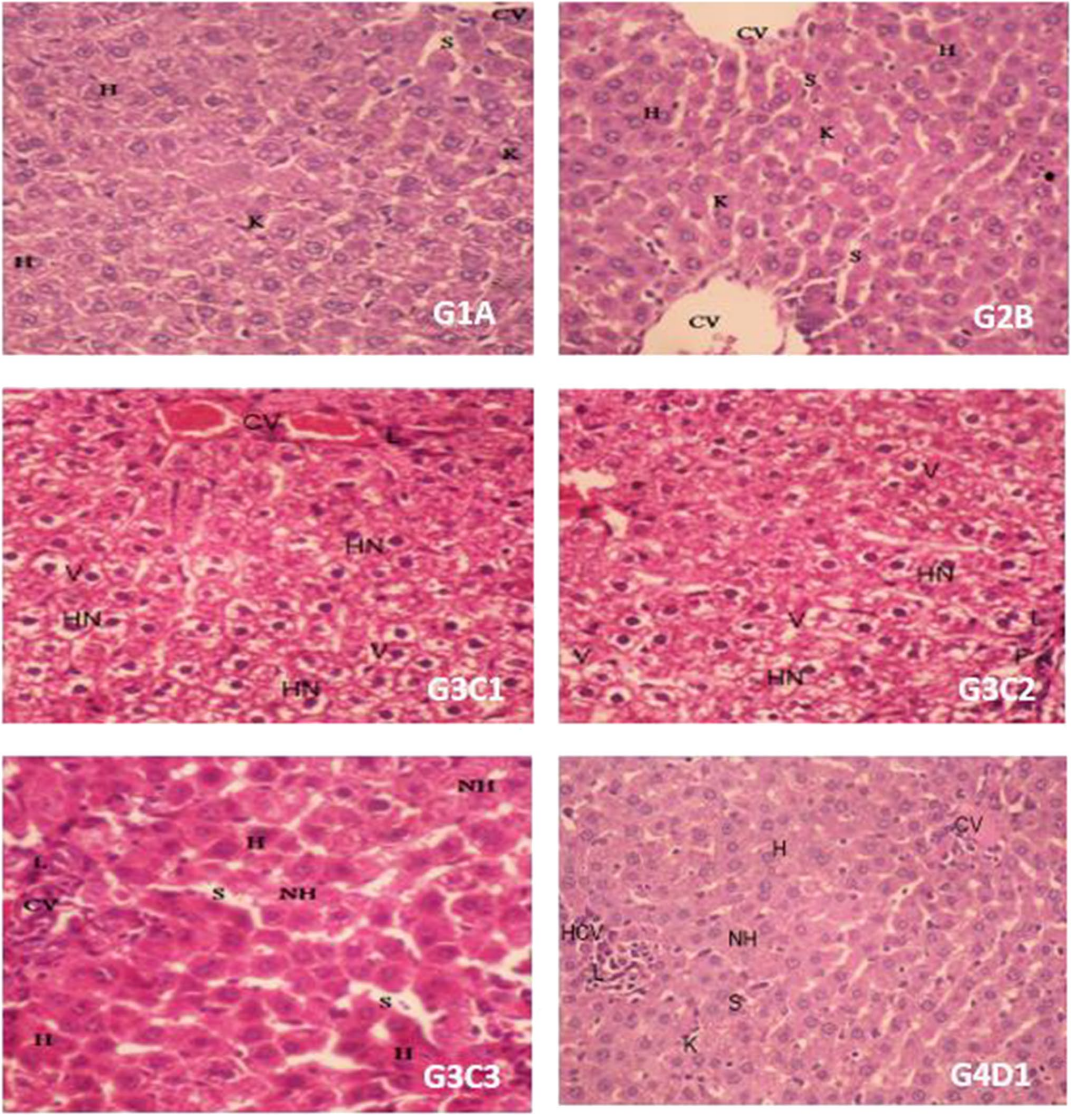
Photomicrograph in rat liver of control (G1A) showing the hepatocytes cells appear as normal architecture with round nuclei containing prominent nucleolus (H), few hepatocytes with homogenous cytoplasm, and sinusoids with studded Kupffer’s cells (K). *E. purpurea* (EP) (G2B) showing marked dilation of central veins (CV). The hepatocytes with large vesiculated nuclei (H), few hepatocytes with homogenous cytoplasm, and an area of crowded hepatocytes with small nuclei. Mild dilation of sinusoids (S) with studded few Kupffer’s cells (K) was seen. Rat treated with CrVI (G3C1) showed two associated and mild dilation of hemorrhage center veins (CV) with few infiltrating lymphocytes (L), crowded and vacuolated hepatocyte cytoplasm (V) with hyperchromatic nuclei (HN). Crowded and vacuolated hepatocyte cytoplasm (V) with hyperchromatic nuclei (HN) are observed. (G3C3) showed moderate dilation of congested two central veins (CV) and infiltrating lymphocytes surrounding the vein (L). Mild dilation of the sinusoid (S) and hyperchromatic with eosinophilic cytoplasm hepatocytes (H) as well as a necrotic one (NH). Rat treated with EP + Cr (VI) (G4D1) showed hemorrhage and mild dilated central vein (CV). The hepatocytes cells appear as normal architecture with round nuclei containing prominent nucleolus (H), few hepatocytes with homogenous cytoplasm with necrotic cell (NH), and mild dilated and hemorrhage sinusoids (S) with studded Kupffer’s cell (K). An aggregated and infiltrating lymphocyte (L) surrounded by crowded hepatocytes was seen (HCV) (H&E × 200)

Histopathological investigations of rats’ liver sections from control (G1A) and EP (G2B) groups showed normal liver structure. The liver is composed of many polygonal lobules and hepatocytes which are separated by irregular sinusoids. In contrast, the liver from CrVI intoxicated rats (G3; C1, C2, and C3) showed distortion in liver architecture as related to the reference group. However, animals that received EP and then were given CrVI dose (G4; D1) demonstrated discernible improvement in the liver structure with crowded hepatocytes and normal nuclei related to the CrVI group (Fig. 3).

### Liver ultrastructure

**Fig. 4:**
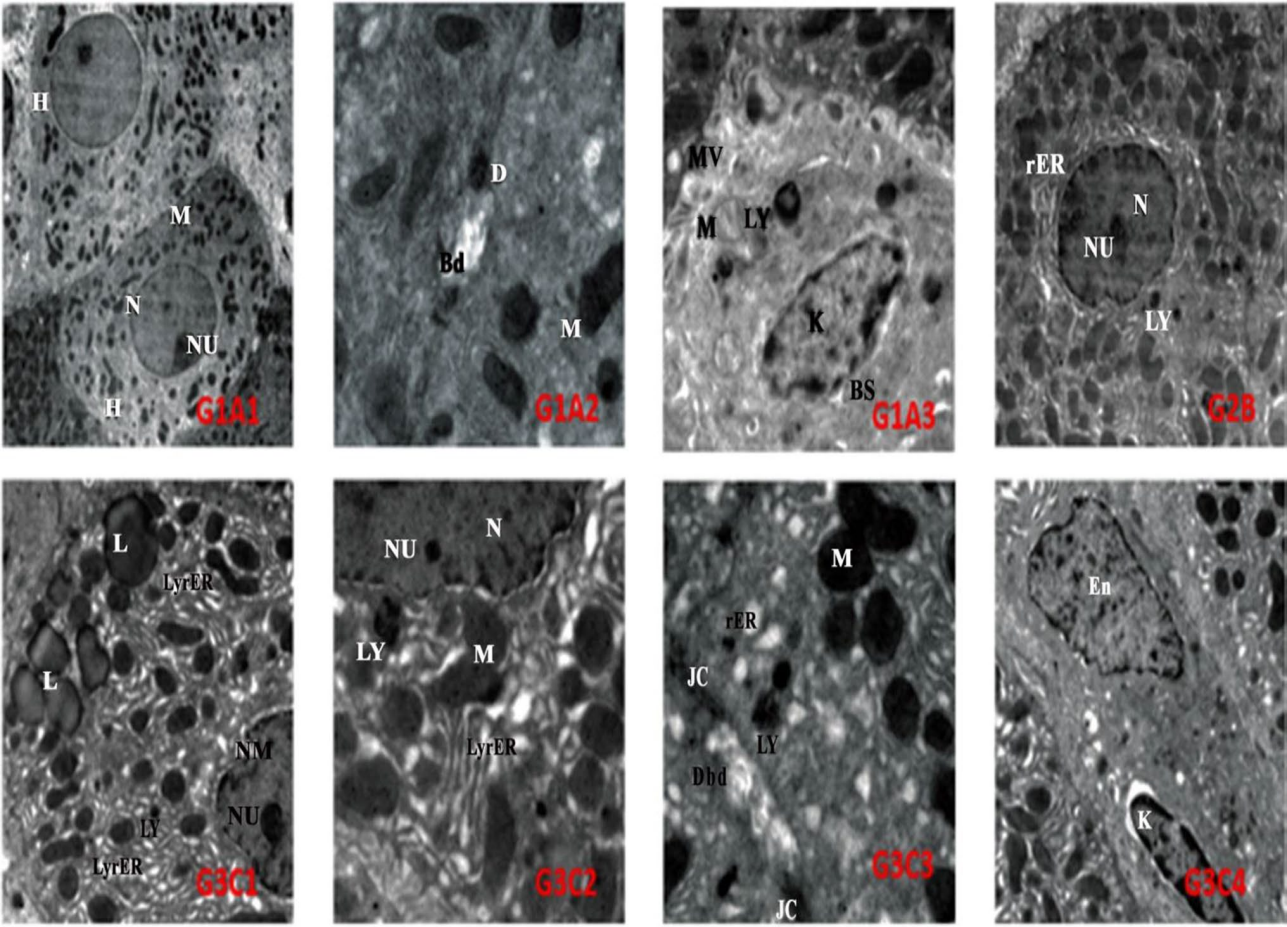
Electron micrographs of the control rat liver (G1A1) show normal hepatocyte (H) with normal nuclei (N) and nucleolus (Nu), mitochondria (M) (scale bar = 5.0 µm, × 1200). (G1A2) illustrating bile duct (Bd) with junctional complexes having desmosomes (D) and condensed configuration mitochondria (M) (scale bar = 1.0 µm, × 5000). (G1A3) showing blood sinusoid (Bs) with kupffer cell (K) and normal mitochondria with normal cristal (M), lysosomes (LY), space of Diss with microvilli (Mv) (scale bar = 1.0 µm, × 5000). Rat hepatocyte treated with *E. purpurea* (EP) (G2B) showing normal hepatocyte with nucleus (N), nucleolus (Nu) surrounded by rER, mitochondria (M), and lysosomes (LY) (scale bar = 2.0µm, × 3000). Rat liver treated with Cr(VI) (G3C1) revealed part of the nucleus with irregular nuclear membrane (Nm), nucleolus (Nu), Lysis of rER (LyrER), lipid (L), and lysosomes (LY) (scale bar = 2.0 µm, × 3000). G3C2 demonstrates part of the nucleus with reduced nucleolus (Nu), mitochondria are condensed configuration (M) lysosomes (LY), and lysis of rER (LyrER) (scale bar = 1.0 µm, × 6000). High power view of a portion of rat liver treated with CrVI (G3C3) revealing damaged bile duct (Dbd), junctional complexes without desmosomes (jc). Lysis of rER, condensed configuration of mitochondria (M), and lysosomes (LY) (scale bar = 1.0 µm, × 6000). Electron micrograph in blood sinusoid of rat liver treated with CrVI (G3C4) showing abnormal endothelial cells (En) and kupffer cells (K) (scale bar = 2.0 µm, × 3000)

**Fig. 5:**
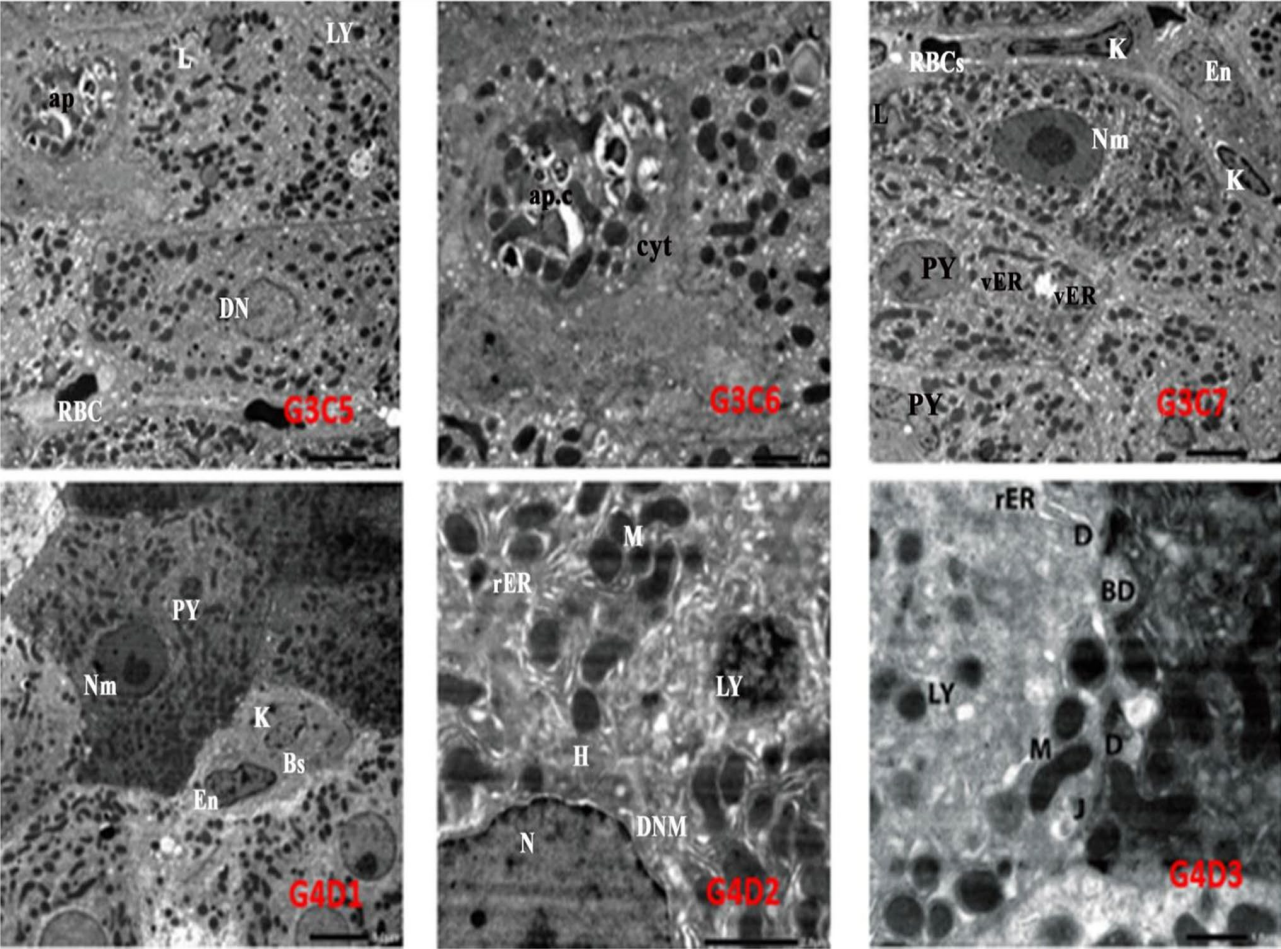
Electron micrograph of rat liver treated with CrVI (G3C5) showing damaged hepatocytes, the cytoplasm without nucleus, and part of cytoplasm comes out surrounded apoptotic body (ap). Other hepatocytes with damaged nucleus (DN), lipids (L), and hemorrhage (RBC) (scale bar = 5.0 µm, × 1000). Electron micrograph of the previous rat liver portion (G3C6) demonstrating apoptotic body surrounded by a part of cytoplasm (cyt) which comes out of the cell forming apoptotic cell (ap.c)/(scale bar = 2.0 µm, × 2000). G3C7 showing damaged liver tissue included pyknosis (pyknotic nuclei) (PY), nuclei with an irregular membrane (Nm), vacuolated (vER), and lipid droplets (L). Blood sinusoid lined by abnormal endothelial cells (En), having activated kupffer cells (K) and RBC (scale bar = 5.0 µm, × 1000). Electron photomicrograph of rat liver received EP then injected with CrVI (G4D1) demonstrating hepatocyte with two nuclei one with slightly irregular nuclear membrane (Nm), and the other is pyknotic (py) and normal organelle, blood sinusoid (Bs) lined by endothelial cell (En) and kupffer cell (K) (scale bar = 5.0 µm, × 1000). Higher magnification of the previous figure; (G4D2) illustrates part of improved hepatocyte (H) with part of normal nucleus (N) with double nuclear membrane (DNM), secondary lysosomes (LY), mitochondria (M), and rER (scale bar = 2.0 µm, × 4000). G4D3 showed bile duct (Bd) and junctional complexes (J) with desmosome (D), lysosomes (LY), mitochondria (M), and rER (scale bar = 1.0 µm, × 5000)

The ultrastructural examination of the control (G1; A1, A2, and A3) and *E. purpurea* (G2B) liver revealed normal hepatocytes with normal nucleus, nucleolus, rER, mitochondria, and lysosomes. While electron micrograph of rat liver treated with CrVI (G3; C1–C7) showed damage in hepatocytes’ cellular constituents. On the other hand, electron photomicrographs of the liver from rats that received EP and then given CrVI (G4; D1, D2, and D3) displayed a marked amendment in the hepatocellular structure as compared to Cr(VI) group (Figs. 4 and 5).

## Discussion

The present investigation was done to assess the possible benefits of *E. purpurea* root extract against CrVI-induced harmful effects in male rats. Oxidative stress was generated by K_2_Cr_2_O_7_ and confirmed by high levels of lipid and protein oxidation markers, and low GSH levels (Halliwell and Gutteridge 2007; Lv et al. 2019). Protein carbonyl groups are mostly formed when proteins’ amino acid side chains are oxidized or when proteins are damaged by ROS (Dalle-Donne et al. 2003). Furthermore, the sulfhydryl groups of cysteine could be converted into their disulfide form via oxidation, leading to a change in the protein redox condition and causing their deactivation (Kuhn et al. 1999). Also, lipid peroxidation is assumed to be predicted by the development of TBARS and H_2_O_2_, which are significant oxidation products (Celik and Suzek 2009). Metal ions are hypothesized to promote lipid peroxidation by contributing to the creation of starting species, increasing peroxidation by breaking down lipid hydroperoxides into various components capable of absorbing hydrogen, and therefore perpetuating the chain reaction of lipid peroxidation (Shahat et al. 2014). Similarly, multiple investigations have found that chromium acts as an oxidant, inducing oxidative stress in different tissues (Marouani et al. 2017; Navya et al. 2018).

Reduced glutathione is implicated in the defense versus ROS and the removal of a variety of poisons. It is renowned that oxidants can produce free radicals by directly reacting with GSH and changing the redox state, or they can release excess free radicals through their metabolism (Maran et al. 2009). The noticed drop in liver GSH might be explained by its role in scavenging free radicals brought on by CrVI leading to its transformation into the oxidized form (GSSG) through metabolism (Chandra et al. 2007; Meister and Anderson 1983). The glutathione reductase enzyme catalyzes the redox cycling of oxidized glutathione, whereas the glucose-6-phosphate dehydrogenase enzyme provides the primary reducing agent, NADPH (Halliwell and Gutteridge 2007). The inhibition of liver antioxidant enzyme activities in rats treated with CrVI may be due to the death of cells expressing or inhibiting these enzymes by ROS. The initial line of defense against oxygen poisoning is thought to be the enzyme catalase and superoxide dismutase (Tan et al. 2018). The superoxide dismutase enzyme spurs the conversion of superoxide anion into H_2_O_2_, which is then reduced by the catalase enzyme into H_2_O, and a decrease in their activities could result in vast radical production (Choudhuri et al. 2020). Furthermore, GSH and GPx are key oxidative stress sensitivity markers. Also, GPx converts peroxide to a harmless molecule to conserve the cellular membrane. Whilst GST is important in the transformation of xenobiotics into nonpoisonous compounds, it also safeguards against oxidative stress and electrophiles (Choudhuri et al. 2020; Ghosh et al. 2012).

Biochemical markers are sensitive indicators of pollution and might be useful diagnostic tools in toxicological research. In the current investigation, CrVI-treated rats had significant changes in liver ALT, AST, ALP, and LDH activities, showing liver cellular injury that affected transport performance and impaired membrane permeability, and enzyme seepage into the bloodstream, pointing out liver toxicity (Chen et al. 2019; AlBasher et al. 2020). The importance of oxidative stress in CrVI-induced liver damage is further supported by the fact that lipid peroxidation performs a critical role in the loss of hepatic integrity of cell membrane that leads to enzyme infiltration (Soudani et al. 2011; Farag and El-Shetry 2020). Alkaline phosphatase, a membrane-bound enzyme with several functions, is employed as a bioindicator of heavy metals poisoning (Ramli et al. 2023). Hepatocellular injury may affect hepatic LDH activity, causing enzyme leakage and compromising the metabolism of carbohydrates and proteins (Zheng et al. 2023). Protein is a vital biological component that can be damaged by free radicals, and its absence could be connected to increased kidney infiltration and/or derangement in protein anabolism and catabolism (Chatterjea and Shinde 2002). Reduced liver absorption, conjugation, or increased bilirubin generation from hemolysis could all contribute to a rise in blood total bilirubin (El-Demerdash 2001). Thus, modifications in these biochemical parameters could be expected as a result of different organ damage (Bashandy et al. 2020).

Hepatocellular damage occurs as a result of toxins decomposition as well as the inflammatory response of stressed hepatocytes. Many protein activities, including NF-κB, are influenced by post-transcriptional alterations like acetylation, methylation, and phosphorylation (Han et al. 2019). Additionally, acetylated NF-κB has high transcriptional activity and stimulates pro-inflammatory cytokines liberation (Zhang et al. 2020). As a result, Yang et al. (2022) speculate that the deacetylation might also be implicated in CrVI hepatotoxicity, finding that CrVI upregulated acetylated NF-κB-p65 level and pro-inflammatory factors IL-1β and TNF-α in the liver by prohibiting Sirtuin 1 deacetylation, leading to an inflammatory response. Furthermore, interleukins activate MAP kinases, a family of protein kinases, at the cellular level. These kinases convey extracellular signals into the nucleus and take part in cellular processes like cell proliferation, differentiation, and apoptosis (Roux and Blenis 2004). For tissue homeostasis and appropriate function, apoptosis is regarded to be essential, and it is crucial in the pathogenesis induced by heavy metals including CrVI (Lu et al. 2018; Zhang et al. 2017). Mitochondrial endogenous apoptosis pathways are regulated by Bax, pro-apoptotic protein and Bcl-2, and anti-apoptotic protein which is induced by oxidative stress and destroyed cellular DNA (Lu et al. 2018). Rats given CrVI treatment in the present study displayed downregulation of Bcl-2 and upregulation of Bax gene expression pointing out that CrVI caused cytotoxicity by inducing the apoptotic process (Navya et al. 2017).

The histological analysis of the livers of chromium-intoxicated rats suggests that the oxidative damage produced by CrVI may have caused significant alterations in liver architecture. Similar findings included tissue and cell damage, morphological changes, DNA damage, chromatin condensation, enlargement and rupture of mitochondria, and even the loss of ridges (Bashandy et al. 2020; Aricthota et al. 2022; Navya et al. 2017). Furthermore, ultrastructural changes in the liver of CrVI-treated rats were consistent with the histology findings where hepatocytes’ nuclei and cytoplasmic organelles both showed these changes. Moreover, the observed mitochondrial damage could lead to ROS liberation, which can lead to oxidative stress and, as a result, cytoplasmic organelle damage. A similar study indicated hepatocyte structural abnormalities, including mitochondrial enlargement, rupture, and even the disappearance of ridges (Yang et al. 2020b).

*Echinacea purpurea* has promising antioxidant capabilities, the majority of which are linked to the high concentrations of bioactive phenolic components (Paudel et al. 2023; Wojdylo et al. 2007; Dogan et al. 2014). However, there are few available findings on the beneficial influences of *E. purpurea* root extract in mammals. By reducing variations in hepatic enzyme activity as well as blood biochemical parameters of the animals, EP root extract displayed a hepatoprotective impact versus the toxic effects of CrVI (Pires et al. 2016; Tsai et al. 2012; Sharif et al. 2021). Furthermore, in comparison to the control group, EP treatment alone reduced oxidative stress indicators and induced liver antioxidant status in rats. However, a significant amendment in PCC, TBARS, and H_2_O_2_ concentrations was observed in rats administered EP plus CrVI. In addition to ROS removal, the noticed augmentation in GSH might shield against protein oxidation through the glutathione redox cycle. Similarly, Matsiopa et al. (2012) found that *Echinacea tincture* significantly increased CAT and GST activities in carbon tetrachloride–treated mice. Also, the rise in the activity of antioxidant enzymes is attributed to the decreased free radicals’ concentration forbidden by EP, which can potentially reduce free radicals’ generation and accumulation. Furthermore, according to Tsai et al. (2012), the antioxidant activity of *E. purpurea* root extract may be due to the plant’s phenolic content and cichoric acid (Hu et al. 2004). Cichoric acid, like flavonoids, exhibits effective radical scavenging ability toward DPPH. While alkamides have not shown any antioxidant activity, they can regenerate cichoric acid by adding hydrogen to the oxidized cichoric acid increasing its activity (Thygesen et al. 2007).

The improvement of tissue thiol pools and the EP’s ability to interact with free radicals or lipid peroxidation active intermediates may help to explain why antioxidant enzymes and glutathione metabolizing enzyme activity are increased while oxidative modification of enzymes is decreased. The most active components of EP such as alkamides and polyacetylenes, caffeic acid derivatives (cichoric acid), melanins, ferulic acid, polysaccharides, and glycoproteins (Luo et al. 2003; Percival 2000; Xu et al. 2021) in addition to chelating substances which are bonded to metals are powerful antioxidants because they minimize redox potency and settle down the oxidized form of metal ions (Shahat et al. 2014). The inhibition of inflammatory cytokine (IL-1β, TNF-α, and NF-κB) activation and release may be a result of EP's antioxidant actions. These results support the Barrett’s work (2003), which showed that *E. purpurea* has anti-inflammatory capabilities. Similarly, the administration of *Curculigo orchoides*, a medicinal plant, reduced the cytotoxicity brought on by CrVI by positively modulating Bcl-2, Bax, and Casp-1 (Navya et al. 2017). Moreover, EP root extract can lessen the changes in the histology and ultrastructure of the liver cells induced by CrVI. Additionally, *E. purpurea* polysaccharide significantly reduced liver dysfunction and pathological changes by keeping the intestinal barrier and modulating liver-related pathways in alcoholic mice livers, (Jiang et al. 2021). In a similar study, Navya et al. (2018) found that *C. orchoides* restored histological changes and enzyme activities in a dose-dependent way. Finally, due to its powerful antioxidant and chelating characteristics, EP root administration significantly lessened tissue injury brought on by acute CrVI toxicity and proved its benefit in reducing oxidative stress.

## Conclusion

Finally, CrVI intoxication resulted in LPO, alterations in the antioxidant defense system, biochemical indicators and genes linked to inflammation, and apoptosis, in addition to histological and ultrastructural changes in the liver. Furthermore, supplementing CrVI-treated rats with EP root extract reduced oxidative damage and recovered the majority of the assessed parameters. As a result, EP had a significant radical scavenging ability by strengthening the antioxidant defense system and reducing free radical formation. Furthermore, our findings support EP’s application as a hepatoprotective nutraceutical.

## Data Availability

The authors declare that all relevant data that support the findings of this study are incorporated in the manuscript.
